# Multi-perspective characterization of seizure prediction based on microstate analysis

**DOI:** 10.3389/fnins.2024.1474782

**Published:** 2024-11-19

**Authors:** Wei Shi, Yina Cao, Fangni Chen, Wei Tong, Lei Zhang, Jian Wan

**Affiliations:** ^1^College of Information and Electronic Engineering, Zhejiang University of Science and Technology, Hangzhou, China; ^2^Zhejiang Key Laboratory of Biomedical Intelligent Computing Technology, Hangzhou, China; ^3^Department of Neurology, The Fourth Affiliated Hospital, International Institutes of Medicine, Zhejiang University School of Medicine, Yiwu, China

**Keywords:** electroencephalogram, seizure prediction, frequency, microstate, nonlinear

## Abstract

Epilepsy is an irregular and recurrent cerebral dysfunction that significantly impacts the affected individual's social functionality and quality of life. This study aims to integrate cognitive dynamic attributes of the brain into seizure prediction, evaluating the effectiveness of various characterization perspectives for seizure prediction, while delving into the impact of varying fragment lengths on the performance of each characterization. We adopted microstate analysis to extract the dynamic properties of cognitive states, calculated the EEG-based and microstate-based features to characterize nonlinear attributes, and assessed the power values across different frequency bands to represent the spectral information of the EEG. Based on the aforementioned characteristics, the predictor achieved a sensitivity of 93.82% on the private FH-ZJU seizure dataset and 93.22% on the Siena Scalp EEG dataset. The study outperforms state-of-the-art works in terms of sensitivity metrics in seizure prediction, indicating that it is crucial to incorporate cognitive dynamic attributes of the brain in seizure prediction.

## 1 Introduction

According to International League Against Epilepsy (ILAE), epilepsy is a chronic cerebral dysfunction resulting from self-limiting abnormal discharges of highly synchronized neurons, often characterized by recurring, episodic, and transient features (Fisher et al., 2014). It frequently arises from birth injuries, traumatic brain injuries, brain infections (e.g., meningitis or encephalitis), and strokes. Seizures typically last from 30 seconds to 2 minutes, followed by a brief interval of confusion and fatigue. Such episodes can lead to secondary harm, even life-threatening injuries, such as drowning or head injuries (Secco, 2020). Consequently, enabling patients to anticipate an impending seizure can significantly mitigate the secondary harm arising from these episodes.

The objective of seizure prediction is to enable patients to anticipate seizures in advance, facilitating the control or prevention of epileptic episodes through medication. Current research in seizure prediction encompasses both long-term and short-term forecasting. Long-term prediction involves investigating the daily and nocturnal cycles, sleep duration, weather patterns, and behavioral factors (Baud et al., 2023; Stirling et al., 2021). On the other hand, short-term prediction involves constant assessment of the risk of impending seizure risk by analyzing data from EEG, magnetoencephalography (MEG), and magnetic resonance imaging (MRI) (Yu et al., 2021). While advancements have been made, there is recognition that improvements are needed, particularly in enhancing the accuracy of long-term predictions and refining the efficacy of short-term warnings. In this study, EEG was chosen for the short-term prediction task due to its cost-effectiveness and ease of data collection.

The essence of short-term seizure prediction lies in distinguishing between interictal(the interval between seizures) and preictal (the period preceding a seizure) states. Upon identifying the current preictal state, a warning is issued. The process of seizure prediction generally involves discerning the differences between preictal and interictal EEG data. Various methodologies are employed to extract relevant features, which are then input into a classifier to achieve prediction outcomes. The features currently used for seizure prediction are often considered from the perspective of nonlinear attributes and spectral information.

Spectral features encompass ample frequency information embedded within EEG data (Usman et al., 2021). These features can be effectively extracted using methods such as short-time Fourier transform (STFT), wavelet transform (WT) or empirical mode decomposition (EMD). Hussein et al. (2021) used an enhanced semi-extended convolutional network and wavelet transformed time-frequency diagrams from raw EEG data to discriminate preictal periods. Wu et al. (2023) highlighted the feasibility of direct end-to-end prediction by employing gamma frequency domain features as input for Long Short-Term Memory (LSTM) models. These studies further demonstrate that diverse seizures in epilepsy are strong correlation between the properties underlying the frequency.

It has been established that a transitional phase precedes seizures, characterized by dynamic fluctuations within the brain system (Le Van Quyen et al., 2001), underscoring the necessity of extracting nonlinear features from EEG data for accurate prediction (Lu et al., 2024). For instance, Mel-Frequency Cepstral Coefficients (MFCCs) were extracted using geometric deep learning (GDL), achieving 95.38% and 96.05% sensitivity on the CHB-MIT and Siena datasets, respectively (Dissanayake et al., 2021). Xu et al. (2022) utilized calculated entropy as input for Gradient Boosting Decision Tree (GBDT) classification, resulting in an accuracy of 91.76%.

However, the current literature offers limited exploration of the significant transformations between interictal and preictal cognitive states, particularly regarding the utilization of their temporal attributes. We hope to leverage this information to improve seizure prediction and gain a deeper understanding of the dynamic alteration mechanisms of the brain during the preictal period.

The brain's cognitive network often exhibits short-term stability, referred to as brain microstates (Koenig et al., 2002). Research has demonstrated that analyzing different microstates and their transitions provides insights into the temporal dynamics of the brain during various cognitive activities or states. This exploration sheds light on the role of dynamic modularity in behavioral control and brain disorders, such as Major Depressive Disorder (MDD) (Zhao et al., 2022; Lei et al., 2022), Attention-Deficit/Hyperactivity Disorder (ADHD) (Luo et al., 2023), Autism Spectrum Disorder (ASD) (Takarae et al., 2022), and Parkinson's disease (PD) (Chu et al., 2023).

Therefore, within the context of these paper, the utilization of microstate analysis becomes imperative to explore the pathological distinctions between the preictal and interictal states, and we will finalize the seizure prediction task by combining nonlinear dynamic changes and spectral changes in preictal.

**Figure 1:**
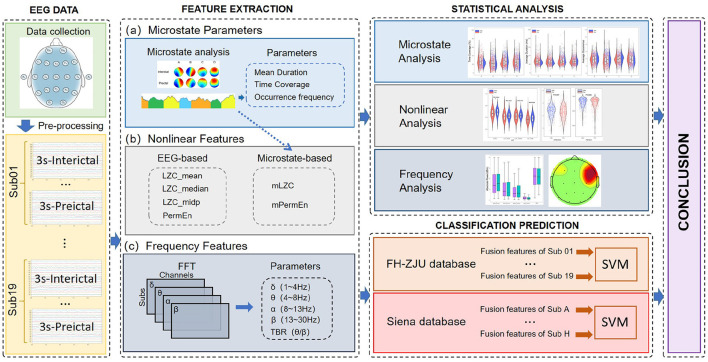
The general flow chart of this paper. It is mainly divided into four parts: EEG data, Feature extraction, Statistical analysis and Classification prediction. The Conclusion learned by the classification prediction and the statistical analysis are combined to explore the seizure pathophysiological mechanisms.

The purpose of this study is to confirm the reliability of cognitive dynamic temporal attributes as biomarkers for epileptic seizures, and investigate how different perspective characterization contribute to seizure prediction across varying data fragment lengths. At the same time, we explored differences of cognitive patterns, nonlinear and spectral properties in preictal and interictal states to assess the underlying pathological mechanisms. As demonstrated in Figure 1, three distinct types of features were extracted in our study, namely microstate parameters (mean duration, time coverage and occurrence frequency), nonlinear features (EEG-based Lempel-Ziv Complexity, Microstate-based Lempel-Ziv Complexity, EEG-based Permutation Entropy, and Microstate-based Permutation Entropy), and spectral features, specifically the absolute power of four frequency bands (δ, θ, α ,β), and Theta-to-Beta Ratio (TBR). Based on these, a comprehensive statistical analysis was performed on both interictal and preictal states, and the seizure prediction task was achieved at the individual-level. Compared with the previous literature, the contributions of this paper are:

1. The cognitive temporal dynamic attribute was first time introduced to forecast seizures, which yielded satisfactory sensitivity, FPR and AUC of 93.82%, 0.061/h and 0.98 on FH-ZJU dataset, and 93.22%, 0.078/h and 0.95 on Siena Scalp EEG dataset, respectively.
2. A microstate-based nonlinear feature (mPermEn) was proposed, which can effectively capture and articulate the random property of cognitive state. The results of the statistical analysis showed that the difference between interictal state and preictal state is more significant for the nonlinear attribute based on cognitive state than for the nonlinear attribute based on EEG data.
3. An in-depth profiling of microstate parameters, nonlinear features, and spectral features was conducted, which compared and analyzed the contribution of seizure prediction from each type of feature. Furthermore, the contribution of each perspective characterization in seizure prediction at different epoch lengths was analyzed.

## 2 Data collection

### 2.1 EEG dataset

This paper has trained and tested the predictor on the FH-ZJU dataset collected from a locate hospital and the Seizure Siena scalp EEG dataset.

The FH-ZJU dataset comprised outpatients from the Department at Neurology of the Fourth Affiliated Hospital of Zhejiang University Medical School between February 2019 and May 2023. The diagnosis adhered to the criteria set by the International League against Epilepsy (ILAE) (Fisher et al., 2014), determined by experienced neurologists using clinical history and continuous video EEG recordings. The clinical continuous EEG recordings utilized in this study were obtained from XLTEK EEG32U system based on the international 10-20 standard (Klem, 1999). During online recording, the impedance of the recording electrode consistently remained below 10KΩ, and the recordings had a bandwidth of 0.05 to 70Hz and a 500Hz sampling rate. Eligibility led to 19 participants (11 males, 8 females, and an average age of 21.84 years). Detailed participant information is shown in Table 1. The study was approved by the Ethics Committee of the Fourth Affiliated Hospital of Zhejiang University School of Medicine and participants provided informed consent.

**Table 1:** Subject information of the FH-ZJU dataset and Siena dataset.

**Dataset**	**ID**	**Age-sex**	**Number**	**Type**	**ID**	**Age-sex**	**Number**	**Type**
FH-ZJU	1	10-F	2	FBTC	11	11-M	1	IAS
	2	12-F	3	IAS	12	15-M	1	WIAS
	3	4-F	1	IAS	13	13-F	2	WIAS
	4	19-M	1	WIAS	14	1-F	2	IAS
	5	31-M	1	IAS	15	68-M	1	WIAS
	6	12-F	3	IAS	16	31-M	2	IAS
	7	4-F	1	IAS	17	16-M	1	WIAS
	8	19-M	1	WIAS	18	18-F	1	IAS
	9	31-M	1	IAS	19	42-F	1	IAS
	10	18-M	1	FBTC				
Siena	A	46-M	2	IAS	G	58-F	1	IAS
	B	54-M	2	IAS	H	71-M	4	IAS
	C	51-F	3	IAS	I	34-F	3	IAS
	D	36-M	5	IAS	J	49-M	4	WIAS
	E	27-F	3	IAS	K	41-F	2	IAS
	F	25-M	9	FBTC				

“Number” presents the number of seizure. “M” means male. “F” means female. “IAS” is focal onset impaired awareness. “WIAS” is focal onset without impaired awareness. “FBTC” is focal to bilateral tonic-clonic.

The Siena Scalp EEG dataset collected by the Unit of Neurology and Neurophysiology at the University of Siena (Detti et al., 2020). It consists of scalp EEG recordings from 14 patients (8 males, 6 females, and an average age of 43.5 years). The recordings were captured with a sampling rate of 512 Hz, with electrodes arranged on the international 10–20 system. Excluding EEG data with excessive channel noise as well as excessive artifacts, we finally remained 11 of the subjects for the experiment. Table 1 reports the details of the selected subjects.

### 2.2 EEG preprocessing

In this study, EEG preprocessing was accomplished using the EEGLAB toolbox (Delorme and Makeig, 2004) in MATLAB (R2021a, MathWorks Inc., USA). The same number of interictal periods equaling in preictal periods per subject was considered to ensure a balanced sample size between subjects, which strongly assure the predictive ability of model. We only used episodes longer than one hours apart to avoid the effects of postictal phase. The preprocessing steps included: (1) Selecting 180-second interictal and preictal EEG segments from each subject; (2) Locating channels based on the international 10–20 system and removing redundant channels like A1 and A2. After removing the useless electrodes, 19 channels remain; (3) Downsampling the data to 250 Hz and implemented a band-stop filter (48 Hz–52 Hz) to mitigate power line interference, as well as a band-pass filter (1 Hz–40 Hz) to remove baseline drift and high-frequency noise, with these filters being second-order Finite Impulse Response filters; (4) Implementing an average reference and performing spline interpolation on channels with peak values exceeding 150 μV for over 20% of data; (5) Employing Independent Component Analysis (ICA) with a 0.9 probability parameter for automatic removal of common artifacts; (6) Segmenting data into 3-second windows with no overlap. The preprocessed data was utilized for subsequent analysis of microstates, nonlinear features computation, and absolute power estimation for each frequency band.

## 3 Methods

In this section, we present the methodology for microstate analysis, the extraction of nonlinear and spectral features, the classification prediction approach, and the statistical analysis procedure.

### 3.1 Microstate parameters

In this research, microstate analysis was performed using the Cartool (Brunet et al., 2011) software, which was divided into five steps: calculating the global field power (GFP), performing clustering at the individual and group levels, back fitting, and extracting features. The detailed process is described below:

The original maps were determined by computing the global field power (GFP) using the following formula:


(1) $$\begin{array}{l} GFP\left(x\right)=\sqrt{\overset{n}{\underset{i=1}{\sum }}{\left[V_{i}-\bar{\text{V}}\right]}^{2}/n}\;,x=1,\cdots \;,N \end{array}$$

where *N* represents the number of sampling points in the sequence, *n* is the number of channels, *V*_*i*_ signifies the value of the *i*-th channel, and $\bar{\text{V}}$ means the average of all channel values. EEG topographic maps around GFP peaks, known for their stable characteristics, were extracted to best represent microstate topology at those instances (Khanna et al., 2015).

To overcome the sensitivity of the improved k-means clustering method to initial values and ensure experimental reproducibility, the Topographic Atomize & Agglomerate Hierarchical Clustering (T-AAHC) method (Von Wegner et al., 2018) was adopted. Using the T-AAHC method, polarity-insensitive clustering analyses were conducted on the original maps with k values ranging from 3 to 8 (Wang et al., 2022). The selection of optimal categories was confirmed using a Meta-Criterion which was discussed in Bréchet et al. (2019), leading to the derivation of the best microstate maps for individuals (Michel and Koenig, 2018).

The optimal individual cluster maps within the same group were used as template graphs for further T-AAHC clustering. Using k = 4, this process yielded the best cluster maps representing group-level characteristics. Four classes of microstate maps were chosen as they have been deemed suitable for describing resting state EEG data and have been frequently used in research on neuropsychiatric disorders.

Group microstate maps were backfitted to individual preprocessed data based on GFP peaks. The data was normalized using the median GFP to mitigate individual scalp potential disparities arising from variations in skull conductivity (Bagdasarov et al., 2022) by calculating non-polarity spatial correlations between group microstate maps and individual EEG topographic maps at GFP peak positions. The microstate with the highest correlation was assigned to a data point, with a minimum correlation threshold of 50%. For smoothing the microstate sequence, segments smaller than 32 ms were halved, with the halves added to the preceding and following segments.

Using the microstate sequence, commonly used parameters (Luo et al., 2023) like average duration (characterizing neuronal stability), time coverage (revealing dominant neuronal patterns), and occurrence frequency (explaining activation tendencies of neurons or the nervous system within each microstate class) were computed.

### 3.2 Nonlinear features

In this section, we computed EEG-based Lempel-Ziv Complexity (LZC) (Lempel and Ziv, 1976), and EEG-based Permutation Entropy (PermEn) (Bandt and Pompe, 2002) to investigate the EEG nonlinear dynamics of both interictal and preictal states. Microstate-based Lempel-Ziv Complexity (mLZC) (Zhao et al., 2022) and Microstate-based Permutation Entropy (mPermEn) are extracted to embody the nonlinear dynamics of cognitive models. All nonlinear features are evaluated using custom scripts in MATLAB.

#### 3.2.1 LZC

LZC is a metric used to assess signal compression through pattern recognition. A lower LZC indicates greater repetition of patterns and lower complexity. This measure finds application in mental and neurological disorders (Ibáñez-Molina et al., 2015).

For EEG discretization, a threshold (Td) is established. Common thresholds include mean, median, and the mean of the minimum and maximum (mid_p) of the EEG sequence:


(2) $$\begin{array}{l} {\text{Td}}_{\text{mean}}=\text{mean}\left({\text{x}}_{1},{\text{x}}_{2},\cdots ,{\text{x}}_{\text{N}}\right) \end{array}$$

(3) $$\begin{array}{l} {\text{Td}}_{\text{median}}=\text{median}\left({\text{x}}_{1},{\text{x}}_{2},\cdots ,{\text{x}}_{\text{N}}\right) \end{array}$$

(4) $$\begin{array}{l} {\text{Td}}_{\text{mid\_p}}=\text{mean}\left(\text{min}\left({\text{x}}_{1},{\text{x}}_{2},\cdots ,{\text{x}}_{\text{N}}\right),\text{max}\left({\text{x}}_{1},{\text{x}}_{2},\cdots ,{\text{x}}_{\text{N}}\right)\right) \end{array}$$

where *N* means the point number of the sequence. The EEG signal is subsequently binary-coded based on the threshold Td :


(5) $$s(i)=\{\begin{array}{l} 0,\text{ if }{\text{x}}_{\text{i}}<\text{Td}\\ 1,\text{ if }{\text{x}}_{\text{i}}\geq \text{Td} \end{array}$$

(6) $$\begin{array}{l} S=\left[s\left(1\right),s\left(2\right),\cdots \;,s\left(i\right),\cdots \;,s\left(N\right)\right] \end{array}$$

where 1 ≤ *i* ≤ *N* and *x*_*i*_ means the EEG data voltage value. After discretization, a sequence *S* composed of 0 and 1 is scanned to count the number of patterns. If a subsequence different from the scanned sequence is encountered, the pattern counter *C*_*w*_ is increased by 1, and the subsequent symbol (0 or 1) indicates the beginning of the next pattern. Upon scanning the complete sequence, LZC is calculated by normalizing *C*_*w*_:


(7) $$\begin{array}{l} \text{LZC}=\frac{{\text{C}}_{\text{w}}}{\text{N}/\log_{2}\text{N}} \end{array}$$

In this study, three types of EEG-based LZC (LZC_mean_, LZC_mid_p_, LZC_median_) are calculated by employing thresholds at the mean, mid_p, and median.

#### 3.2.2 mLZC

LZC quantifies the repetition of EEG signals patterns by summarizing scalp voltage values, but it may not fully reflect changes in deep brain function. To address this, we integrate LZC with microstates, yielding mLZC, which directly represents repeatability among thought atomic while also resisting noise interference. Based on microstate analysis, a microstate sequence can be formulated:


(8) $$\begin{array}{l} MS=\left[ms\left(1\right),ms\left(2\right),\cdots \;,ms\left(i\right),\cdots \;,ms\left(N\right)\right] \end{array}$$

where *N* means the number of sampling points; *ms*(*i*) ∈ {1, 2, 3, 4} represents microstate A, B, C and D, respectively. We calculated the number of incompressible pattern to characterize the repeatability of this microstate sequence. The calculation process is outlined in Algorithm 1:

**Algorithm 1:**
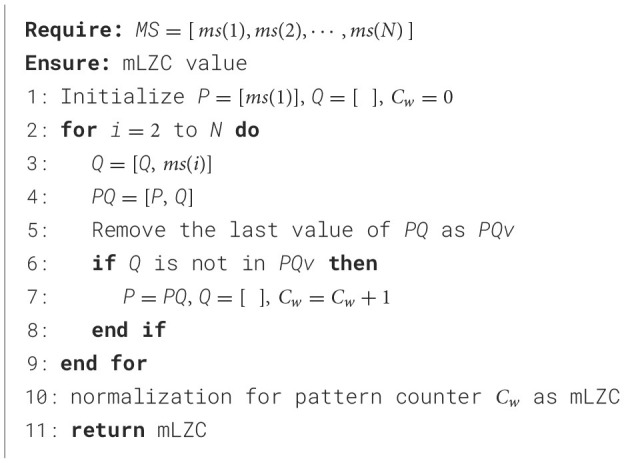
mLZC Calculation Algorithm.

#### 3.2.3 PermEn

Permutation entropy serves as a specialized metric for quantifying time series random. This is achieved by generating distinct permutation modes through comparisons with neighboring data points. The computation entails the following steps:

Initially, the time delay τ and the embedding dimension *m* are established using the approach in Ibáñez-Molina et al. (2015), which to draw upon Recurrence Quantification Analysis (RQA).

The next step involves the reconstruction of the feature space. This entails arranging the component vector of the reconstruction matrix in ascending order of value. This step assists in identifying the pattern within the pattern group composed of *m*. The reconstruction matrix is following:


(9) $$\text{R}=\left[\begin{array}{cccc} x(\text{1}) & x(1+\tau ) & \cdots & x(1+(m-1)\tau )\\ \vdots & \vdots & & \vdots \\ x(k) & x(k+\tau ) & \cdots & x(k+(m-1)\tau )\\ \vdots & \vdots & & \vdots \\ x(K) & x(K+\tau ) & \cdots & x(K+(m-1)\tau ) \end{array}\right]$$

where *K* = *N*−(*m*−1)τ; *k* = 1, 2, ⋯ , *K* and *x*(*k*) denotes the EEG data voltage value. This procedure generates a pattern sequence, which encapsulates the patterns identified within the dataset, as shown:


(10) $$\begin{array}{l} T=\left[t\left(1\right),t\left(2\right),\cdots \;,t\left(k\right),\cdots \;,t\left(K\right)\right] \end{array}$$

where *t*(*k*)∈{1, 2, ⋯ , *m*!} and *t*(*k*) indicates the sequence number that matches the arrangement pattern. Subsequently, the Shannon entropy formula is applied as follows:


(11) $$\begin{array}{l} H_{p}=-\overset{m!}{\underset{l=1}{\sum }}p_{l}\ln\;p_{l} \end{array}$$

where *p*_*l*_ denotes the probability of each permutation pattern occurring and *l* ∈ {1, 2, ⋯ , *m*!}. Finally, the permutation entropy is estimated using the aforementioned formula. This metric provides valuable insights into quantifying the inherent complexity within the time series data.


(12) $$\begin{array}{l} \text{PermEn}=\frac{{\text{H}}_{\text{p}}}{\ln\left(\text{m}!\right)} \end{array}$$

#### 3.2.4 mPermEn

Permutation entropy can only reveal the randomness of brain discharge activities by detecting the randomness and dynamic displacement in time series, which has not been much researched on the deep-seated functional dynamic changes of the brain. In this study, we proposed a feature to characterize microstate randomness, and calculate a index, mPermEn, by using the probability distribution of the permutation pattern of each microstate subsequence. It effectively captures the randomness of microstate permutation patterns, representing the complexity of brain cognitive changes.

There is a phenomenon of sampling points belonging to the same microstate class for a period of time, which can seriously affect the probability distribution of several permutation patterns. Therefore, before calculating mPermEn, we removed several sampling points adjacent to the same microstate class in the microstate sequence (8). A new sequence of microstates is obtained:


(13) $$\begin{array}{l} MSv=\left[ms\left(1\right),ms\left(2\right),\cdots \;,ms\left(u\right),\cdots \;,ms\left(U\right)\right] \end{array}$$

where *ms*(*u*) ≠ *ms*(*u* − 1), *ms*(*u*) ≠ *ms*(*u* + 1) and *U* ≤ *N*. We set the embedding dimension *m* to be the same as the number of microstate classes, and keep the time delay τ consistent with PermEn calculation. Therefore, we can attain a reconstruction matrix R as (9). And we also construct a *m*^*m*^ × *m* dimensional permutation pattern matrix X and a zero vector of 1 × *m*^*m*^ dimensional as index vector Y:


(14) $$\begin{array}{l} \text{X}={\left[\begin{array}{cccc} a_{11} & a_{12} & \cdots & a_{1m}\\ a_{21} & a_{22} & \cdots & a_{2m}\\ a_{31} & a_{32} & \cdots & a_{3m}\\ \vdots & \vdots & \vdots \\ a_{m^{m}1} & a_{m^{m}2} & \cdots & a_{m^{m}m} \end{array}\right]}_{{\text{m}}^{\text{m}}\times \text{m}} \end{array}$$

(15) $$\begin{array}{l} \text{Y}={\left[0,0,\cdots \;,0\right]}_{1\times {\text{m}}^{\text{m}}} \end{array}$$

where $a_{jh}=\left\lceil j/m^{m-h}\right\rceil \;\mathrm{mod}\;m$, *j* ∈ {1, 2, ⋯ , *m*^*m*^}, *h* ∈ {1, 2, ⋯ , *m*}. Each row of the reconstructed matrix R is then compared with each row of the permutation pattern matrix X to determine which permutation pattern the reconstructed matrix R belongs to, and add 1 to the corresponding position of the index vector Y.

Finally, calculate the probability distribution for each permutation pattern and obtain mPermEn using the Equations 11, 12. The calculation process of mPermEn is outlined in Algorithm 2:

**Algorithm 2:**
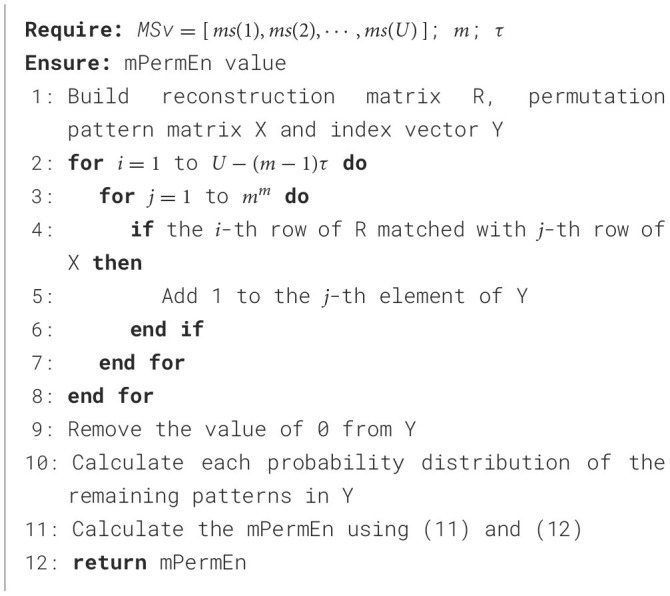
mPermEn Calculate Algorithm.

### 3.3 Spectral features

In this study, absolute power was computed for four distinct frequency bands: δ (1–4 Hz), θ (4–8 Hz), α (8–12 Hz), and β (12–30 Hz), along with the TBR, which is commonly used in neurological disease assessment (Luo et al., 2023; Group et al., 2023). The spectral analysis was carried out in two sequential steps. The initial step aimed to identify significant differences in frequency bands between the preictal and interictal states. Subsequently, the analysis sought to identify channels exhibiting significant differences within the specific frequency bands.

The power of each segment was computed using FFT, followed by the summation of power within each frequency band to yield the power of that band. Averaging across subjects and channels yielded a matrix of dimensions N_group_ * N_subj_ * N_fre_, where N_group_ represents interictal and preictal groups, N_subj_ signifies the number of subjects, and N_fre_ corresponds to the count of frequency bands and TBR. By statistically analyzing the power matrix of these groups, differential frequency bands between the interictal and preictal periods were identified.

Once a distinct frequency band was ascertained, recalculating the absolute power within that band resulted in a matrix of dimensions N_group_ * N_subj_ * N_channel_, where N_channel_ refers to the count of EEG recording electrodes. Statistical analysis of this power matrix across groups highlighted channels exhibiting frequency band-specific differences.

### 3.4 Predictive classification

For the seizure prediction task, we undertook both individual-level classifications over the normalized feature set.

In the individual-level classification task, we focused on classifying each subject's data into two states. To avoid evaluation bias caused by model overfitting, we first set aside 10% of the data as the test set, while the remaining 90% is used for training and validation. We applied 5-fold cross-validation to partition the training and validation sets, and employed grid search to optimize the hyperparameters of the support vector machine. This entire process was repeated 10 times, and the final performance evaluation was based on the average performance of the test set over the 10 iterations.

We primarily quantified classifier performance using metrics such as accuracy, sensitivity, false positive rate (FPR), and the area under the curve (AUC). In traditional classification tasks, accuracy is often considered a key performance metric. However, in this study, detecting the preictal class is critical due to its lower occurrence compared to the interictal class. As the preictal class is treated as the positive class, sensitivity becomes more important than specificity and accuracy in evaluating the proposed method. FPR represents the average number of false seizure predictions per hour of EEG recording, calculated as the number of predicted seizure events within 1 hour that do not overlap with actual reference seizures. AUC was used to assess the overall classifier performance, with higher values indicating better classification and discrimination.

In addition, to evaluate the classification effectiveness across each feature category, we separately inputted microstate parameters, nonlinear features, and spectral features into the SVM classifier. In the end, there are epochs of multiple lengths extracted for seizure prediction to explore the effect of data length on microstate parameters, nonlinear features and spectral features.

### 3.5 Statistical analysis

In this study, the R (v4.3.0) (Rahlf, 2017) was used for statistical analyses to identify and elucidate features with significant differences between the interictal and preictal states. Initially, the Kruskal-Wallis test was performed across clusters of microstate topographic maps to identify variations between the interictal and preictal conditions. Subsequently, the Shapiro-Wilk normal distribution test was performed on the each feature. Paired samples t-test was conducted for data that met the normal distribution, otherwise Wilcoxon signed rank sum test was used. Throughout the experiment, a trend emerged where the significance of outcomes increased with an increase in sample size. This phenomenon results in, with a substantial sample size, the significance indicated by the α level of 0.05 deviates from the real-world context. To address this, the G*Power software (Faul et al., 2009) was used to adjust the α significance level. The determination of α was guided by the desired effect size, power, and the extant sample size, ensuring that it accurately and scientifically mirrored the real-world significance. Cohen'sd effect size (Terpou et al., 2022) was also adopted to quantitatively express the distinction between the interictal and preictal states.

## 4 Results and discussions

### 4.1 Prediction performance

We compiled the microstate parameters, nonlinear features, and spectral features into a consolidated feature matrix, which was then utilized as input for the SVM classifier. The results yielded promising individual-level classification metrics, including an average accuracy of 94.80%, sensitivity of 93.82%, FPR of 0.061/h, and AUC of 0.98 on FH-ZJU dataset; accuracy of 92.32%, sensitivity of 93.22%, FPR of 0.078/h, and AUC of 0.95 on Siena Scalp EEG dataset.

Table 2 portrays a comparison of the performance of existing methods of predicting seizures with scalp EEG dataset in recent years. At present, the most widely used dataset for seizure prediction is the CHB-MIT dataset (Lu et al., 2023a), whose EEG dataset of patients with medically uncontrollable seizures was collected by the Children's Hospital Boston in collaboration with the Massachusetts Institute of Technology and contains 22 subjects (5 males and 17 females). It can be seen from the table, majority of the researches achieved a sensitivity of 91% or more, implying that their methods are good at seizure prediction. One of the more notable performances is the method of Usman et al. (2021) and Lu et al. (2023b). Fusing the automatic features extracted by convolutional neural network (CNN) with manually extracted temporal features, Usman et al. (2021) trained using SVM, CNN and LSTM respectively, and achieved a sensitivity of 96.28% over CHB-MIT dataset. Lu et al. (2023b) used 3D CNN model to automatically extract information from EEG signals after STFT and later used Bi-LSTM for classification. Finally, a sensitivity of 98.40% and an accuracy of 97.95% were achieved on the CHB-MIT dataset. Both of them achieve a excellent performance by automatically extracting features using CNNs, however, the involved perspectives embracing time or spectral attributions are slightly single.

**Table 2:** Comparison of existing individual-level seizure prediction methods using scalp EEG signals.

**Dataset**	**Num**	**Reference**	**Accuracy (%)**	**Sensitive (%)**	**FPR (/h)**	**AUC**
CHB-MIT dataset	22	(Zhang et al., 2019)	90.00	92.20	0.120	0.90
	13	(Yang et al., 2021)	92.07	89.33	-	0.91
	22	(Usman et al., 2021)	-	96.28	-	-
	12	(Gao et al., 2022)	-	94.60	0.060	0.94
	13	(Wu et al., 2023)	-	91.76	0.290	-
	11	(Lu et al., 2023b)	97.95	98.40	0.017	-
Siena dataset	12	(Jiang et al., 2023)	85.71	83.00	-	-
	13	(Kapoor et al., 2022)	95.31	93.18	-	-
	11	this work	92.32	93.22	0.078	0.95
FH-ZJU	19	this work	94.80	93.82	0.061	0.98

“Num” presents the number of subjects. The symbol “-” represents that the author did not use the indicator during forecasting.

Since the microstate analysis method used in this paper requires channel localization for single-lead EEG data. Therefore, we tested the proposed method on the Siena Scalp EEG dataset and compared it with previous works using the same dataset. Jiang et al. (2023) proposed a method based on frequency-domain analysis and phase-amplitude coupling (PAC) combined with a random forest classifier for seizure prediction and ultimately achieved an accuracy of 85.71% and a sensitivity of 83% on Siena EEG dataset. Kapoor et al. (2022) used a seizure prediction algorithm consisting of hybrid optimization control integrated classifier to classify wavelet and entropy based features in each frequency band and finally achieved accuracy of 95.31% and sensitivity of 93.18% on Siena dataset. Similar to Jiang et al. (2023) and Kapoor et al. (2022), this study does not employ a data-driven prediction approach. Instead, it bases predictions on the features that exhibit the most significant differences between the interictal and preictal periods. However, unlike these previous works, this study not only incorporates spectral information and nonlinear properties but also integrates the microstate temporal dynamics properties, achieving higher sensitivity.

**Figure 2:**
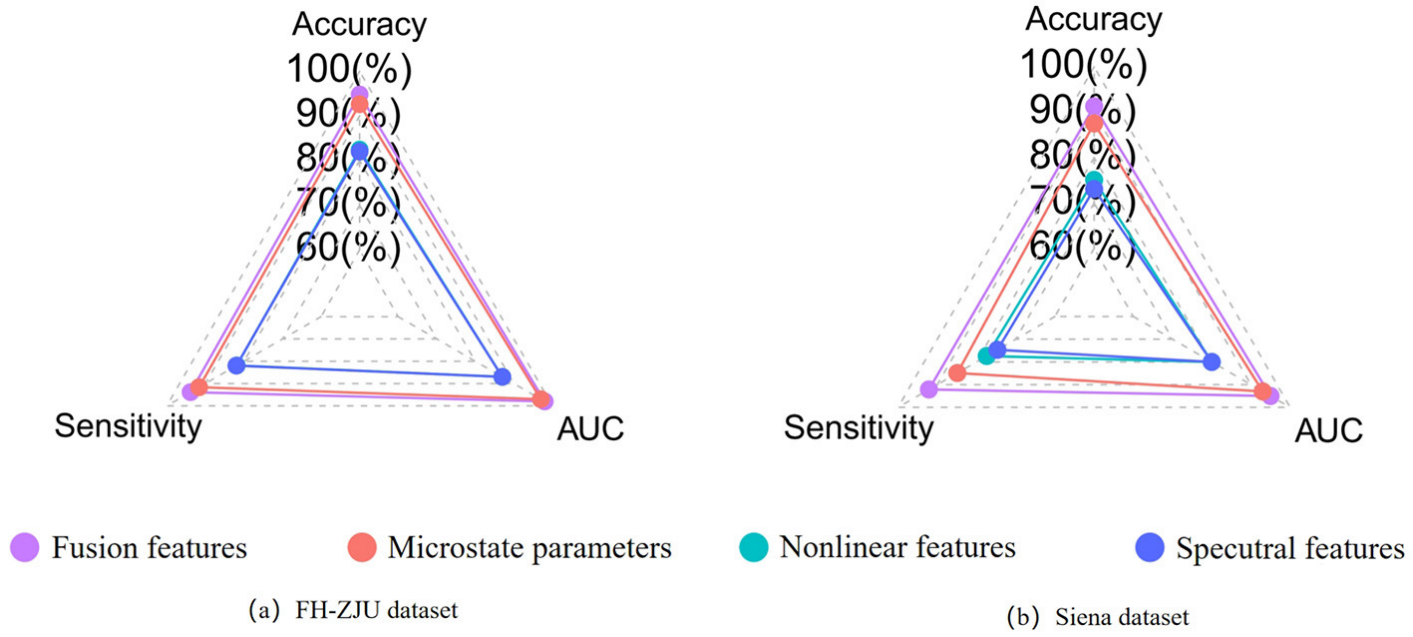
**(a)** Comparison of the individual-level performance of multiple types of features on the FHZJU dataset. **(b)** Comparison of the individual-level performance of multiple types of features on the Siena dataset.

To further assess the predictive efficacy of different feature types, we subjected microstate parameters, nonlinear features, spectral features, and their combined fusion features (referred to as “fusion features”) to classification at the individual level. The results as displayed in Table 3. Besides, to better compare the classification performance between different features visually, the results are shown in the radar plot of Figure 2. The figure shows that whether in the FH-ZJU dataset or the Siena dataset, it is with the same trend. Apart from fusion features, microstate features demonstrating the most pronounced classification performance, followed by nonlinear features and spectral features. The accuracy of fusion features and microstate parameters was more than 10% higher than that of nonlinear and spectral features across both the FH-ZJU and Siena datasets. Besides, the accuracy of fusion and microstate features was quite similar, with differences of only 2.18% on the FH-ZJU dataset and 3.79% on the Siena dataset. It highlights that the fusion feature matrix primarily derives its potency from microstate features. This means that the microstate temporal dynamic properties of EEG data are more suitable for seizure prediction tasks than nonlinear and spectral information. Besides, it can be seen from the performance of fusion features that the complementary information from multiple feature types can significantly improve the prediction performance.

**Table 3:** Individual-level predictive performance results for multiple types of features.

**Dataset**	**Feature**	**Accuracy (%)**	**Sensitive (%)**	**FPR (/h)**	**AUC**	**Kappa**
FH-ZJU dataset	Fusion-Features	94.80	93.82	0.061	0.98	89.48
	Microstate	92.62	91.61	0.083	0.97	84.38
	Nonlinear	82.45	81.88	0.181	0.87	63.15
	Spectrum	81.99	81.96	0.180	0.87	62.42
Siena dataset	Fusion-Features	92.32	93.22	0.078	0.95	82.50
	Microstate	87.53	84.89	0.151	0.93	74.96
	Nonlinear	75.10	77.39	0.226	0.80	59.28
	Spectrum	73.09	74.66	0.253	0.80	55.72

Kappa: Cohen's Kappa is a statistical measure of the degree of agreement between the classifier output and the clinical labels.

**Figure 3:**
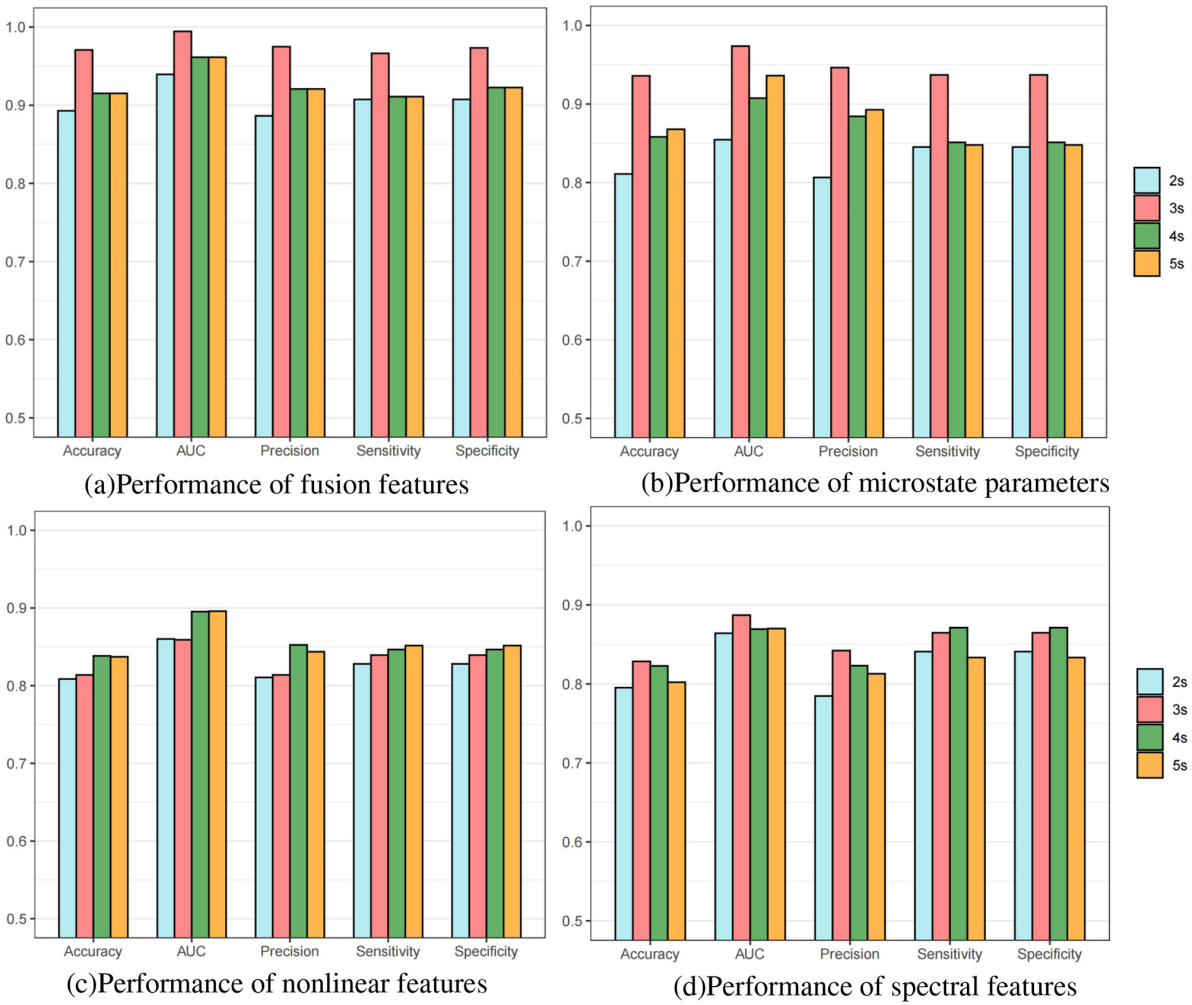
Bar charts of individual-level predictive performance of different length epochs on FH-ZJU dataset. **(a)** Performance of fusion features. **(b)** Performance of microstate parameters. **(c)** Performance of nonlinear features. **(d)** Performance of spectral features.

Furthermore, we conducted subject-individual prediction experiments with different data lengths on the FH-ZJU dataset to verify the influence of data lengths on these features. From Figure 3a, it is seen that 3s data length have the optimum performance, 4s and 5s had a similar performance between that of 3 s and 2 s. To explore why the fundamental reason for the optimal performance of 3s data length, we separately conducted experiments on each features with different lengths at the individual-level. The results of microstate parameters, nonlinear features and spectral features are depicted respectively in Figures 3b–d. Comparing the four bar charts, we found that the distribution of microstate parameters in Figure 3b is nearly close to that of fusion features in Figure 3a, which reveals that the advantage of fusion features with 3s data length mainly comes from microstate features with 3s data length. These results gave a further verification on the significance of microstate temporal dynamic properties in seizure prediction. From Figures 3c, d, we can see that the results of different fragment lengths of nonlinear features and spectral features hold steady, which indicated that the nonlinear features and spectral features are less sensitive to the data length.

Notably, despite using a basic SVM model in this experiment, the prediction performance was remarkably high. This outcome can likely be attributed to two key factors. First, the dataset used in this study contained relatively few seizure events, and SVMs are well-suited for binary classification tasks with small sample sizes, allowing the model to maintain reliable prediction accuracy even with limited data. Second, the length of the preictal prediction window also significantly impacts the model's performance (Lu et al., 2021). Longer prediction windows may provide more advanced warnings but could introduce additional noise and uncertainty, while shorter windows may improve prediction accuracy by focusing on more immediate preictal changes. Therefore, optimizing the length of the prediction window is crucial for further enhancing the model's predictive capabilities.

### 4.2 Microstate properties between interictal and preictal

**Figure 4:**
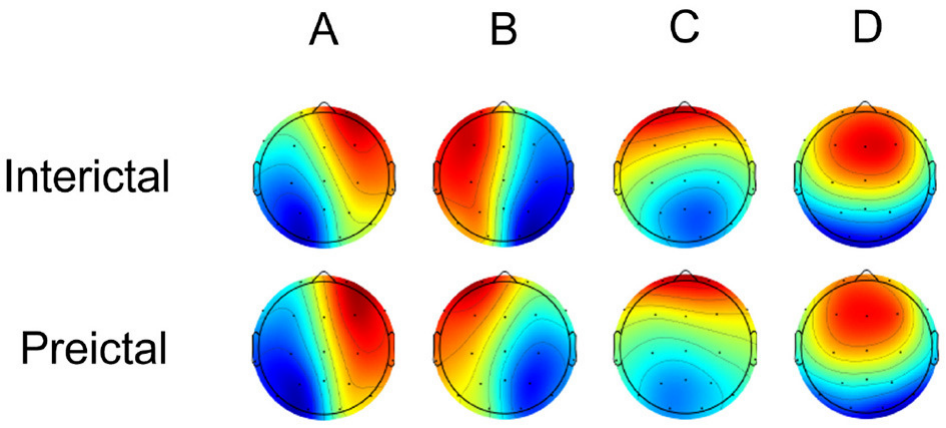
Four canonical microstate topographic maps of Interictal and Preictal.

Microstate analysis of scalp electroencephalogram in the interictal and preictal groups yielded four canonical microstate topographic maps, as shown in Figure 4. These maps are consistent with those reported in previous studies on neurological disorders and are characterized as follows: class A (right-frontal and left-posterior topographies), class B (left-frontal and right-posterior topographies), class C (midline and frontal-occipital topographies), and class D (midline and frontal topographies). The four canonical microstate structures, often aligned with inherent brain functional networks (class A linked to the auditory network, class B to the visual imagination network, class C to the salience network, and class D to the attention network), exhibit general consistency between interictal and preictal states. The microstate maps showed overall consistency between the interictal and preictal states, accounting for 76.11% and 78.12% of the global explained variance (GEV) in the interictal and preictal groups, respectively. Consistent with Takarae et al. (2022), Kruskal-Wallis tests indicated no statistically significant differences between the interictal and preictal groups for any of the microstate maps [p(A) = 0.78, p(B) = 0.53, p(C) = 0.92, p(D) = 0.80].

Three parameters were obtained to capture the dynamic shifts in thought atom by applying microstate map backfitting to EEG data. We set the effect size, the power, and the sample size to 0.3, 0.95 and 600, and the adjustment of α was adjusted to 0.0005 using G*Power software. The test results and Cohen'sd value highlighted significant alterations in microstate dynamics between the interictal and preictal states, as depicted in Table 4. We thought that only when the P-value is less than 0.0005 and the absolute value of Cohen'sd exceeded 0.3, there are significant difference between preictal and interictal. The findings revealed that during the preictal phase, class A exhibited markedly elevated duration and coverage compared to its interictal counterpart. Conversely, class C displayed considerably reduced duration and coverage relative to interictal observations. Furthermore, the analysis of occurrence characteristics revealed that class A and class B microstates were lower in preictal states than interictal states, while occurrences of class D microstates were higher in preictal states than interictal states. Based on these phenomena, we hypothesize that seizures may result from changes that make microstate C more likely to shift to microstate A. We will continue to explore this point in our next work.

**Table 4:** Statistics of microstate parameters between interictal and preictal states.

**Parameter**	**Type**	***P*-value**	**Cohen'sd**
Duration	Class A	**4.62e-15** ^***^	**-0.4508**
	Class B	0.0425	-0.1154
	Class C	**3.54e-11** ^***^	**0.3796**
	Class D	0.2600	0.0640
Coverage	Class A	**4.59e-17** ^***^	**-0.4839**
	Class B	0.0018	0.1775
	Class C	**2.16e-15** ^***^	**0.4564**
	Class D	0.0031	-0.1685
Occurrence	Class A	**3.58e-05** ^*^	0.2356
	Class B	**2.57-07** ^***^	0.2943
	Class C	0.1375	0.0844
	Class D	**7.51e-05** ^*^	-0.2256

P-Value: ^*^ for p < 5e-4, ^***^ for p < 1e-6.

Cohen'sd: Black bold means Cohen'sd < -0.3 or Cohen'sd>0.3.

### 4.3 Nonlinear attributes between interictal and preictal

**Figure 5:**
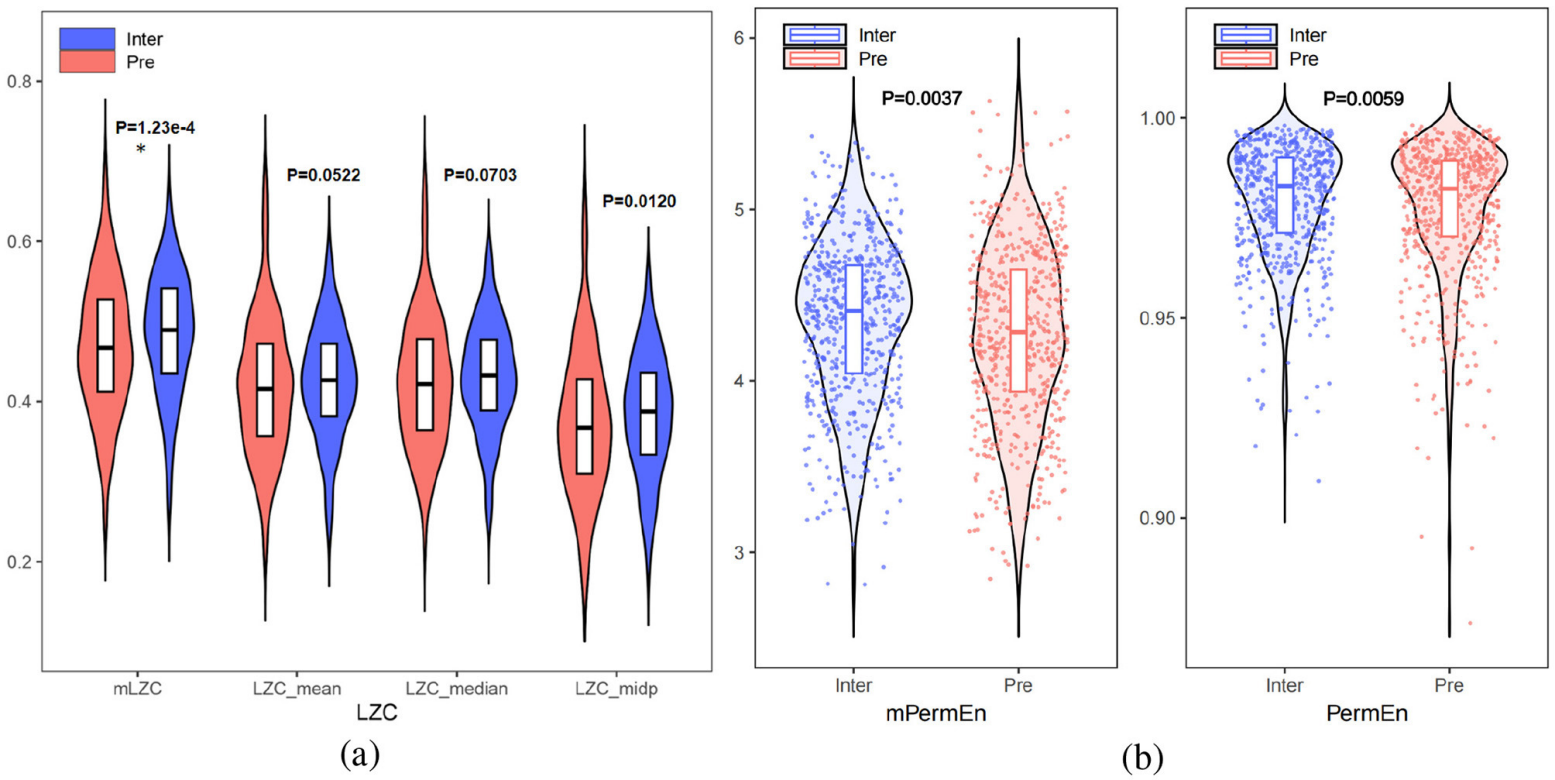
**(a)** Comparison of LZC_mean_, LZC_median_ LZC_mid_p_ with mLZC between interictal and preictal. **(b)** Comparison of PermEn and mPermEn between interictal and preictal (^*^ for *p* <5e-4).

To quantify the nonlinear dynamic changes between interictal and preictal states, six nonlinear features were extracted: mLZC, LZC_mean_, LZC_median_, LZC_mid_p_, mPermEn, and PermEn. As shown in Figures 5a, b, the test revealed that preictal complexity generally exhibited lower values compared to interictal complexity, along with greater numerical dispersion, which was consistent with Lehnertz (2008) as well as Babloyantz and Destexhe (1986), suggesting that epileptogenicity involves complexity reduction. Seizure activity is characterized by weakened nonlinearity, whereas interictal EEG demonstrates higher nonlinearity. Furthermore, we hypothesize that synchronized abnormal neuron firing seizures leads to this reduction in nonlinearity. In the 3 minutes preceding a seizure, nonlinearity displayed a decreasing trend, indicating a gradual loss of autonomous brain function. Additionally, individual EEG variations in the preictal state were greater than those in the interictal state, where complexity values tended to cluster within a normal range.

As shown in Figures 5a, b, the proposed microstate-based features, mPermEn (*p* = 0.0037) and mLZC (*p* = 1.23e-4), more effectively distinguished between interictal and preictal states compared to the EEG-based features PermEn (*p* = 0.0059), LZC_mean_ (*p* = 0.0522), LZC_median_ (*p* = 0.0703), and LZC_mid_p_ (*p* = 0.0120). This highlights that mPermEn and mLZC aptly capture the complexity changes in brain cognitive activity, going beyond the traditional exploration of epilepsy pathology based solely on EEG signal complexity.

### 4.4 Spectral information between interictal and preictal

**Figure 6:**
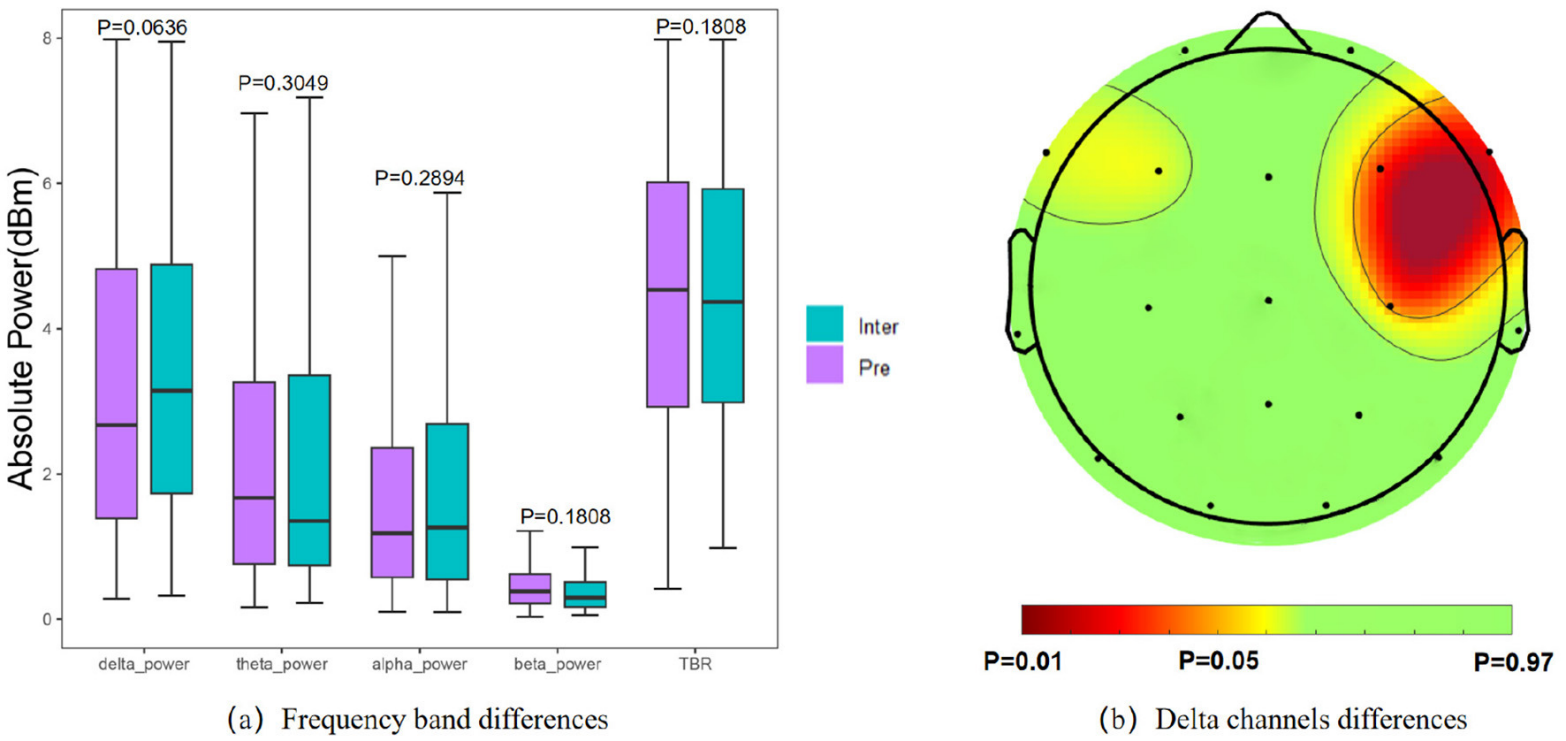
Results of statistical analysis of spectral features. **(a)** Comparison of power in each frequency band. **(b)** Significantly different of the δ band power on each channel.

For statistical analysis of spectral characteristics, we calculated the power of each frequency band and adjusted the significance level α to 0.05. The paired t-test results indicated no significant difference in absolute power across frequency bands between interictal and preictal states (Figure 6a). However, upon closer examination, the δ bands showed decreased power in preictal states, while θ band power and TBR exhibited an increasing trend prior to seizures. Among the frequency bands, the δ band displayed the most substantial variation between the interictal and preictal states. We further analyzed differences in the δ band across different channels (Figure 6b), revealing significant disparities in the right frontal brain regions, specifically in the F4 (*p* = 0.0203), C4 (*p* = 0.0372), and F8 (*p* = 0.0113) channels.

A comparison of the statistical analysis results of the microstate parameters, nonlinear features and spectral features reveals that the microstate parameters exhibited the most significant differences between interictal and preictal phases, followed by the nonlinear features, which aligns with the predictive results. These findings underscore that epileptic seizures result in abnormal changes in brain microstate dynamics.

## 5 Conclusion

In this paper, we employed cognitive dynamic attributes to delineate the preictal state. By integrating nonlinear and spectral characteristics, a remarkable performance was achieved on both the FH-ZJU and Siena dataset. Besides, we proposed a novel microstate-based nonlinear feature, mPermEn, to effectively capture and articulate the randomness at the microstate level between interictal and preictal states. Comparative analysis revealed that changes in cognitive pattern dynamics using microstates better characterized the preictal period than nonlinear attributes and spectral information. Moreover, microstate properties within 3-second fragments proved more suitable for seizure prediction tasks. In all, microstate analysis not only elucidates temporal dynamic alterations in neurological diseases but also serves as a foundational approach for other EEG analysis methods, offering substantial potential for exploring the brain's cognitive mechanisms.

## 6 Limitation

The research method employed in this article has certain limitations. Specifically, relying solely on LZC, permutation entropy, and power as nonlinear and spectral features limits our ability to comprehensively capture such information. Consequently, our findings only preliminarily suggest that cognitive dynamic attributes may be superior to nonlinear and spectral metrics in characterizing the preictal phase. Moving forward, we will use additional, more representative features in these perspective characterization.

As the aim of this article is to introduce brain cognitive dynamic attributes into seizure prediction, assessing their potential as seizure biomarkers. To this end, we chose a basic SVM classifier rather than more intricate models. In future work, we plan to explore complex models to enhance seizure prediction accuracy.

## Data Availability

The datasets presented in this article are not readily available because part of the data involves patient privacy. Requests to access the datasets should be directed to Fangni Chen, chenfangni@zust.edu.cn.
